# Field Performance and Diagnostic Accuracy of a Low-Cost Instrument-Free Point-of-Care CD4 Test (Visitect CD4) Performed by Different Health Worker Cadres among Pregnant Women

**DOI:** 10.1128/JCM.01277-18

**Published:** 2019-01-30

**Authors:** Stanley Luchters, Karl Technau, Yasmin Mohamed, Matthew F. Chersich, Paul A. Agius, Minh D. Pham, Mary L. Garcia, James Forbes, Andrew Shepherd, Ashraf Coovadia, Suzanne M. Crowe, David A. Anderson

**Affiliations:** a Burnet Institute, Melbourne, Victoria, Australia; b Department of Epidemiology and Preventive Medicine, Monash University, Melbourne, Victoria, Australia; c International Centre for Reproductive Health, Department of Public Health and Primary Care, Ghent University, Ghent, Belgium; d Empilweni Services and Research Unit, Department of Paediatrics & Child Health, Rahima Moosa Mother and Child Hospital, Faculty of Health Sciences, University of the Witwatersrand, Johannesburg, South Africa; e Wits Reproductive Health and HIV Institute, Faculty of Health Sciences, University of the Witwatersrand, Johannesburg, South Africa; f Omega Diagnostics, Ltd., Omega House, Alva, Scotland; g The Alfred Hospital and Department of Infectious Diseases, Monash University, Melbourne, Victoria, Australia; Rhode Island Hospital

**Keywords:** CD4 count, field performance, HIV, South Africa, diagnostic accuracy, point-of-care diagnostics, sensitivity, specificity, task shifting

## Abstract

Measuring CD4 counts remains an important component of HIV care. The Visitect CD4 is the first instrument-free low-cost point-of-care CD4 test with results interpreted visually after 40 min, providing a result of ≥350 CD4 cells/mm^3^. The field performance and diagnostic accuracy of the test was assessed among HIV-infected pregnant women in South Africa. A nurse performed testing at the point-of-care using both venous and finger-prick blood, and a counselor and laboratory staff tested venous blood in the clinic laboratory (four Visitect CD4 tests/participant). Performance was compared to the mean CD4 count from duplicate flow cytometry tests on venous blood (FACSCalibur Trucount). In 2017, 156 patients were enrolled, providing a total of 624 Visitect CD4 tests (468 venous and 156 finger-prick samples). Of 624 tests, 28 (4.5%) were inconclusive. Generalized linear mixed modeling showed better performance of the test on venous blood (sensitivity = 81.7%; 95% confidence interval [CI] = 72.3 to 91.1]; specificity = 82.6%, 95% CI = 77.1 to 88.1) than on finger-prick specimens (sensitivity = 60.7%; 95% CI = 45.0 to 76.3; specificity = 89.5%, 95% CI = 83.2 to 95.8; *P* = 0.001). No difference in performance was detected by cadre of health worker (*P* = 0.113) or between point-of-care versus laboratory-based testing (*P* = 0.108). Adequate performance of Visitect CD4 with different operators and at the point of care, with no need of electricity or instrument, shows the potential utility of this device, especially for facilitating decentralization of CD4 testing services in rural areas.

## Abstract (precis)

Measuring CD4 counts remains an important component of HIV care. The Visitect CD4 is the first instrument-free low-cost point-of-care CD4 test with results interpreted visually after 40 min, providing a result of ≥350 CD4 cells/mm^3^.

## INTRODUCTION

Since the earliest days of HIV treatment and care, measuring CD4 cell counts has been an important component of the continuum of care for adults and children living with HIV. The CD4 count is an accurate predictor of disease status and immediate risk of death and, as such, CD4 count testing at baseline for all people living with HIV remains recommended (1, 2). CD4 testing continues to be used to assess eligibility for elements of a package of care, including tracking disease progression, monitoring the effectiveness of antiretroviral treatment (ART), adapting adherence support, antimicrobial prophylaxis and preemptive treatment, screening for opportunistic infections, and vaccination management, as well as ART prioritization (2, 3).

Recommendations for ART initiation based on the CD4 level have evolved over time, with World Health Organization (WHO) guidelines arguing for a move to 350 cells/mm^3^ in 2010, a move to 500 cells/mm^3^ in 2013 (4, 5), and then the removal of the threshold altogether in 2015 (6). Regardless of the epidemic profile and disease burden, patients with a CD4 count below 350 cells/mm^3^ should be prioritized for ART and care (7). This group is at high risk of mortality, opportunistic infections, and cancers related to HIV. CD4 count level is therefore still used for prioritizing ART and fast-tracking patients with more advanced disease (2, 8).

Where ART is not immediately initiated, CD4 cell count assessments are recommended every 6 to 12 months (7). In settings where viral load testing is not routinely available (around half of ART patients worldwide), the WHO recommends that a CD4 count, in conjunction with clinical monitoring, should be used to diagnose treatment failure (3). Moreover, patients with unstable or advanced HIV disease require ongoing CD4 monitoring, regardless of whether viral load testing is available (2, 6).

Despite wide variability and poor precision of CD4 count assessments (9–11), flow cytometric methods have provided the gold standard for CD4 monitoring. These assays require an electronic instrument, typically with constant power or batteries, and regular equipment maintenance, as well as highly trained technicians and cold-chain management of reagents (12, 13). Such requirements have restricted access to testing for patients distant from central laboratories or well-established clinics. Availability of point-of-care CD4 tests such as the Alere Pima CD4 has facilitated immediate decision-making, patient management, and referral and improved patient care and retention in some settings (14). Although performance of point-of-care CD4 technologies has been acceptable (9, 15), implementation challenges remained due to the need for powered instrumentation, device failures, maintenance requirements, and operator errors (15).

The Burnet Institute (Melbourne, Australia) has developed a low-cost (ca. $6.00 [U.S. currency]/test) disposable instrument-free point-of-care CD4 test (Visitect CD4; licensed to Omega Diagnostics, United Kingdom). The test is based on a lateral flow principle, detecting the amount of T-cell-associated CD4 antigen, and gives visual semiquantitative results (above or below 350 CD4 cells/mm^3^) after 40 min. We sought to assess here the field performance and diagnostic accuracy of the Visitect CD4 test as performed by different health worker cadres among HIV-infected pregnant women.

## MATERIALS AND METHODS

### Study design.

This prospective diagnostic accuracy study with cross-sectional design is reported according to the STARD Statement for Reporting Studies of Diagnostic Accuracy (16). Performance of the Visitect CD4 was assessed using venous and finger-prick blood and performed by a variety of intended users either at the clinical laboratory or at the point of care.

### Participants.

Pregnant women living with HIV aged 18 years and older and who were attending antenatal services at Rahima Moosa Mother and Child Hospital in Johannesburg were recruited consecutively and invited to participate. To be eligible, women needed to have confirmed HIV infection, as documented by two separate HIV rapid antibody tests, and provide written informed consent. Women received only the result of the flow cytometry test.

### Study procedures.

Participants completed a brief questionnaire on sociodemographic characteristics, gestational age, and health-seeking variables. Two types of blood samples were obtained from patients in the antenatal clinic: a finger-prick specimen taken by the nurse and a venous blood sample (in two 5-ml EDTA tubes) obtained by a phlebotomist.

### CD4 testing.

Six CD4 tests were performed in parallel: (i) Visitect CD4 from finger-prick blood by a nurse at the point of care (one test); (ii) Visitect CD4 from venous blood by a nurse at the point of care (one test); (iii) Visitect CD4 from venous blood at the clinic laboratory (two tests, performed by a counselor, laboratory technologist, or other trained health worker); and (iv) a flow cytometric CD4 cell count from venous blood at the reference laboratory (two tests). All EDTA blood specimens were analyzed within 12 h of venipuncture. Operators and readers of the tests were blinded to the results of other tests and the clinical status of patients (aside from HIV status).

### Reference test.

The flow cytometry reference tests were performed with the same venous sample used in the clinic laboratory and tested on the same day by qualified laboratory personnel at BioAnalytical Research Corporation (BARC), a SANAS-accredited research laboratory in Johannesburg, South Africa, using a BD FACSCalibur Trucount flow cytometry machine (Becton Dickinson, San Jose, CA). A regular external quality assurance (EQA) program was performed on the reference test equipment through the UK National EQA Service. The mean value between the two flow cytometry measurements was taken as the reference value.

### Visitect CD4 test.

**FIG 1:**
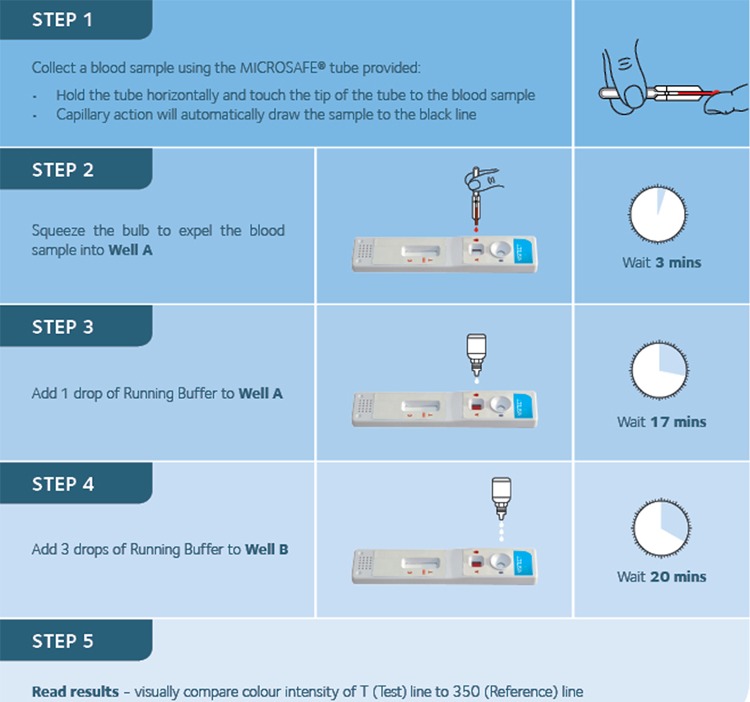
Timing and procedures for each step of the Visitect CD4 test.

Visitect CD4 test kits contain a disposable test device, a 30-µl micropipette for finger-prick blood collection, one bottle of buffer, a sterile alcohol swab, and a safety lancet. Additional materials required to run the test include disposable gloves, a timer, and equipment for the safe disposal of used lancets. The Visitect CD4 test was performed according to the test procedure outlined in Fig. 1. Finger-prick blood was obtained using the provided safety lancets, 30 µl of blood was then collected in the micropipette and added to well A. Venous blood was collected according to standard hospital procedures using EDTA blood tubes. These were opened at point of care, and 30 µl was removed using a Gilson pipette and similarly added to well A. At 3 min after the addition of the blood sample to the test device, one drop of running buffer was added to well A. After a 17-min wait, three drops of buffer were added to well B. The test result was then read visually after another 20 min, and no more than 25 min (i.e., no more than 45 min after test commencement). Tests read after this time were categorized as inconclusive.

The Visitect CD4 test is a semiquantitative test with results classified as either >350 cells/mm^3^ or ≤350 cells/mm^3^. Tests results are visually interpreted by comparing the intensity of the test line with the reference line: if the test line is darker than the reference line, the result is >350 cells/mm^3^; if the test line is lighter than the reference line, then the result is ≤350 cells/mm^3^. Invalid tests without a control line present were also considered inconclusive.

The first of the two Visitect CD4 tests performed on venous blood in the clinic laboratory was read twice visually by independent operators; all other tests were read once by the respective operator. All four Visitect CD4 tests performed for each study participant were also scanned using an Axxin AX-2 reader to provide a permanent record of the test results, but only visually interpreted data were used for analysis. Visitect CD4 tests used came from one of eight manufactured pilot batches; these were manufactured with the same raw material specifications and using manufacturing process similar to that used for the final product which gained CE marking in November 2017. The used tests had an expiration date approximately 1 month after the last participant was assessed.

### Visitect CD4 training.

Study staff and Visitect CD4 operators, including nurses, counselors, one phlebotomist, and one laboratory technician, initially attended a 3-day training (total, 10 h) in preparation for an earlier beta testing study of 68 participants (data not shown). The training covered study objectives and design, study procedures, recruitment, consent, confidentiality, and the logistics of implementation. Training days 2 and 3 were conducted by two staff from Omega Diagnostics, focused on developing the knowledge and skills to accurately perform the Visitect CD4 test, and included collection of finger-prick blood samples, the transfer of blood using micropipettes, the correct application of buffer, and visual interpretation of the test results. Before commencement of this study, an additional 2-day refresher training (5 h) and supervision of the testing took place in early 2017, led by Omega Diagnostics. Training focused largely on the test procedures and data collection processes and included several one-on-one sessions and supervision of the tests. A *Proficiency Testing Guide* was used to facilitate and standardize competency assessment. Role plays were used to ensure understanding of the informed consent and questionnaire administration procedures.

### Data management and analysis.

Study data were collected and managed using REDCap (research electronic data capture) electronic data capture tools hosted at the University of the Witwatersrand, which is a secure, password-protected, web-based application designed to support data capture for research studies (17). All data were entered once, and each entry was checked for accuracy and was only available to staff directly involved in data entry or analysis.

Data were obtained on patient ages, gestational ages, whether the patients were receiving ART, and out-of-pocket costs to participants associated with that particular visit, which included transport to and from the clinic, as well as incidental costs such as childcare. Data were also collected on the time taken from phlebotomy/finger-prick to testing and the time taken between steps for each of the Visitect CD4 tests.

Pooled performance (sensitivity, specificity, positive predictive values [PPV], and negative predictive values [NPV]) results are presented separately for venous and finger-prick samples according to STARD guidelines (16). Moreover, the pooled performance of valid Visitect CD4 tests (i.e., excluding invalid Visitect CD4 tests and Visitect CD4 tests that were not performed according to the testing protocol) with conclusive reference tests, which were performed by either a nurse, counselor, or laboratory staff, was examined using generalized linear mixed modeling (GLMM; generalized through logit link function and binomial distribution). For sensitivity and specificity estimation, GLMM was used given the dependency in test data (i.e., a single sample was tested separately by different operators) where study participant positivity on the Visitect CD4 test was treated as a random effect, and fixed terms for the reference test, operator, blood sample type, and their interactions were also estimated to explore the extent to which these factors moderate test performance. Nested likelihood ratio tests were used to provide inference in comparing unadjusted and unconstrained models (i.e., exploration of the differential performance across blood sample types and operators), and Bonferroni-corrected postestimation linear-equation Wald tests were used to provide inference for further *post hoc* comparison across operator levels. Marginal probabilities integrated over the estimated distribution of the random effect (18), and 95% confidence intervals (CI) from GLMM were produced to estimate the sensitivity and specificity for respective models. For PPV and NPV estimation, marginal probabilities from pooled GLMM analyses with Huber/white cluster robust sandwich variance estimation was used since GLMM analyses exhibited convergence inconsistencies (19). Performance was assessed at an intended cutoff of ≤350 cells/mm^3^. A separate performance assessment was done for the same test at ≤200 cells/mm^3^ to assess the ability of Visitect CD4 to identify those with more advanced HIV disease.

Visitect CD4 test reproducibility (i.e., device variability) and interobserver reliability were also assessed. One venous blood Visitect CD4 test was read by two independent health workers, who were blinded to the test results of the other to assess interobserver reliability, using the κ statistic, including 95% CI values. Test reproducibility was also assessed by comparing the diagnostic performance of the three main operator cadres (nurses, counselor, and laboratory staff) of the Visitect CD4 tests on each study participant using GLMM, as described above.

The reproducibility of the reference flow cytometry CD4 level measurement was examined by estimating the 95% limits of agreement between the two tests and also visually by plotting person-specific differences in measurement versus the mean CD4 level (20). In addition, to further quantify the level of agreement between flow cytometry CD4 measurements, variance components modeling of flow cytometry CD4 measurements and derivations based on within-subject mean bias and variance in CD4 measurement (21) were undertaken to provide both intraclass correlation coefficient (ICC) and coefficient of variation (CV) estimates, respectively.

All analyses were undertaken using Stata version 14.2, and statistical significance was assessed at the 5% level.

This study was approved by the University of Witwatersrand Human Research Ethics Committee (medical; protocol reference M140534) and the Alfred Hospital Ethics Committee, Australia (project 256/14).

## RESULTS

### Participant characteristics.

At Rahima Moosa Mother and Child Hospital, 156 patients were enrolled between March and June 2017. Participants were a median 33 years of age (interquartile range [IQR], 28.5 to 36; range, 19 to 46). At the time of testing, 5.8, 26.3, and 66.0% of women were in their first, second, or third trimester of pregnancy, respectively, and less than half (42.3%) had completed four or more antenatal care visits. The large majority of the women (98.7%) were receiving antiretroviral therapy. One in ten women (16/156) reported to have missed or delayed an antenatal visit because of costs of these visits. The women reported spending a median $1.9 per visit for transport and incidentals to the clinic (IQR, $1.5 to $2.3; range, $0.7 to $11.4).

### Reference test results.

**FIG 2:**
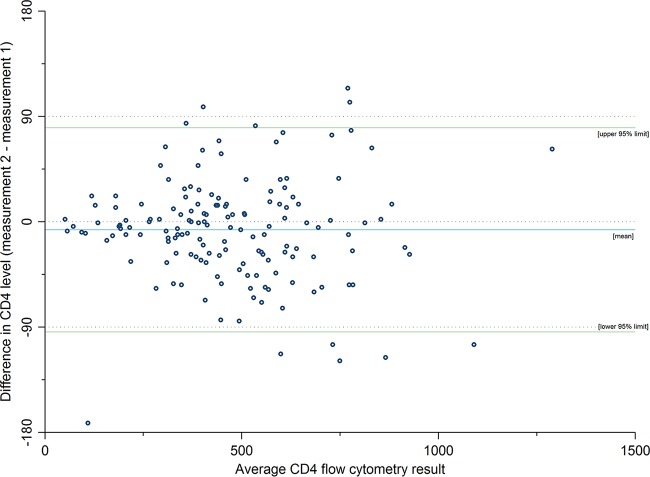
Difference in flow cytometry CD4 measurements across the two test occasions by mean CD4 level among 153 participants.

**FIG 3:**
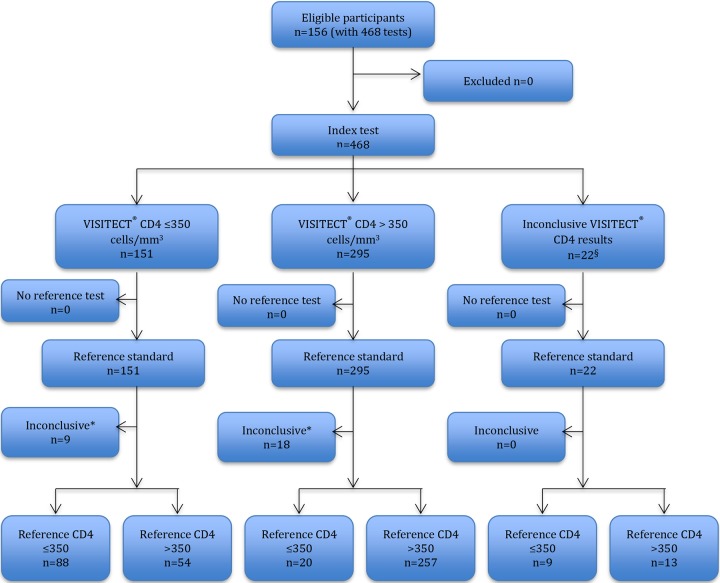
Flow diagram of 468 Visitect CD4 tests performed on 156 venous EDTA samples (pooled analysis from all operators) and reported in accordance with the STARD statement (16).

**FIG 4:**
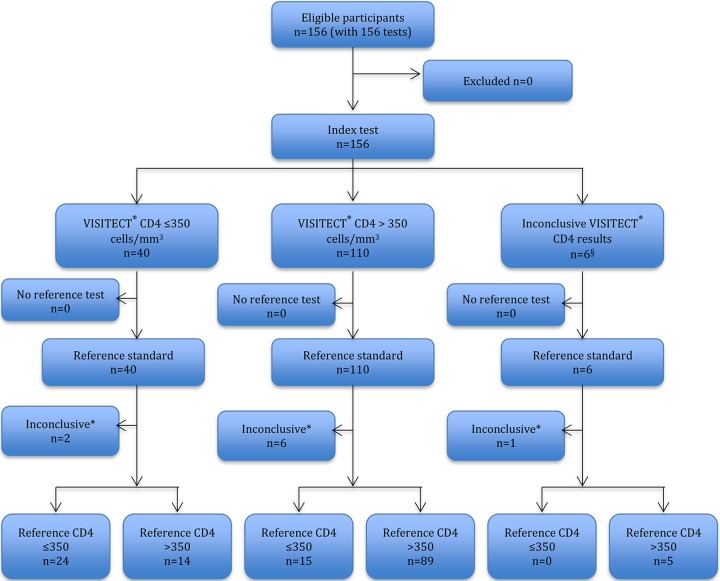
Flow diagram of 156 Visitect CD4 tests done on 156 finger-prick EDTA samples performed by the nurse and reported in accordance with the STARD statement (16).

Two participants had only a single reference test result, and one had no reference result (see Fig. 3 and 4). Six participants (3.9%) had discrepant reference CD4 cell counts of around 350 cells/mm^3^ cutoff (i.e., reference tests above and below the 350-cells/mm^3^ cutoff). The remaining 147 participants with duplicate reference test results had a mean CD4 cell count of 478 cells/mm^3^ (standard deviations [SD] = 215), with 39 women (26.5%) having a CD4 cell count of ≤350 cells/mm^3^ and 108 (73.5%) having >350 cells/mm^3^ (Fig. 2).

The mean difference (or bias) between reference CD4 test assessments from 153 participants with duplicate assessments was –6.9 cells/mm^3^ (SD = 44.5; Fig. 2). The 95% limits of agreement between the two assessments ranged from –94.2 to 80.3 cells/mm^3^. The intraclass correlation coefficient for participant flow cytometry CD4 measurements was high (ICC = 0.98, 95% CI = 0.97 to 0.98), and the coefficient of variation was approximately 15% (CV = 14.7%, 95% CI = 12.9 to 16.5%).

### Visitect CD4 field performance and diagnostic accuracy.

A total of 624 Visitect CD4 tests were performed (three venous and one finger prick). Visitect CD4 performance is visually presented in flow diagrams in accordance with STARD guidelines (16) separately for the 468 tests on venous EDTA samples (Fig. 3) and 156 tests on finger-prick samples (Fig. 4).

Of 624 Visitect CD4 tests, 28 (4.5%) were inconclusive: 22 inconclusive results of 468 venous samples (5 invalid results and 17 operator failures) and 6 inconclusive results of 156 finger-prick samples (3 invalid results and 3 operator failures). Operator failures involved tests that were not performed according to the test protocol, as self-reported by the respective operator, and were all read more than 45 min after test commencement.

Our GLMM performed on the 147 women with 539 conclusive reference tests and conclusive Visitect CD4 tests (of 561 tests, there were 22 tests performed by other operators and excluded from GLMM) showed a statistically significant difference in the diagnostic performance of the Visitect CD4 when performed on venous blood compared to finger-prick blood [LR χ^2^(2) = 14.32, *P* = 0.001; Table 1, model C]. Performance on venous blood at CD4 350 mm^3^ cutoff showed a sensitivity of 81.7% (95% CI = 72.3 to 91.1) and specificity of 82.6% (95% CI = 77.1 to 88.1), whereas the diagnostic performance on finger-prick blood showed a sensitivity of 60.7% (95% CI = 45.0 to 76.3) and specificity of 89.5% (95% CI = 83.2 to 95.8).

**TABLE 1:** Diagnostic accuracy of Visitect CD4 at a cutoff of ≤350 cells/mm^3^ from GLMM^*a*^

Factor	Value (95% CI)												Inference^*b*^ and heterogeneity		
	Model A				Model B				Model C						
	Sens	Spec	PPV	NPV	Sens	Spec	PPV	NPV	Sens	Spec	PPV	NPV	LR (χ^2^ )	*P*	ICC
Overall	75.9 (66.1–85.8)	84.3 (79.3–89.3)	64.7 (53.1–76.3)	90.5 (85.7–95.3)											0.57
															
Operator													χ^2^(4) = 7.48	0.113	0.58
Lab tech					80.6 (67.9–93.3)	84.8 (77.8–91.8)	66.7 (52.8–80.5)	92.4 (87.0–97.8)							
Nurse					70.5 (58.3–82.7)	86.7 (81.2–92.2)	67.7 (54.3–80.8)	88.4 (82.5–94.3)							
Counselor					82.4 (70.1–94.8)	79.7 (72.0–87.4)	58.8 (45.3–72.4)	93.1 (87.8–98.4)							
															
Sample type													χ^2^(2) = 14.32	0.001	0.61
Venous									81.7 (72.3–91.1)	82.6 (77.1–88.1)	63.0 (51.3–74.7)	92.6 (88.0–97.1)			
Finger-prick									60.7 (45.0–76.3)	89.5 (83.2–95.8)	71.9 (56.2–87.5)	85.0 (78.0–92.0)			

a GLMM generalized through use of a logit link function and binomial distribution with a random intercept for test participant (*n* = 147). Sens, test sensitivity; Spec, test specificity; PPV, positive predictive value; NPV, negative predictive value; ICC, intraclass correlation coefficient from random intercept GLMM; model A, unadjusted; model B, operator by test interaction and main effect (not shown); model C, Blood sample type by test interaction and main effect (not shown). PPV and NPV estimates were determined from ordinary logit GLM with cluster robust standard errors. GLMM analyses would not converge reliably.

b Likelihood ratio (LR) tests comparing nested less-constrained models (B and C) with model A.

The Visitect CD4 test showed higher sensitivity in diagnosing those with more advanced HIV immune suppression (Table 2). On venous blood, diagnostic performance of Visitect CD4 for CD4 ≤ 200 cells/mm^3^ showed a sensitivity of 91.1% (95% CI = 80.4 to 100) and a specificity of 73.1% (95% CI = 66.9 to 79.3). On finger-prick blood, the sensitivity was 80.2% (95% CI = 62.0 to 98.3) and the specificity was 83.4% (95% CI = 76.8 to 90.1; Table 2, model C), and values differed significantly from venous blood performance [LR χ^2^(2) = 12.31, *P* = 0.002].

**TABLE 2:** Diagnostic accuracy of Visitect CD4 at a cutoff of ≤200 cells/mm^3^ from GLMM^*a*^

Factor	Value (95% CI)												Inference^*b*^ and heterogeneity		
	Model A				Model B				Model C						
	Sens	Spec	PPV	NPV	Sens	Spec	PPV	NPV	Sens	Spec	PPV	NPV	LR (χ^2^)	*P*	ICC
Overall	87.9 (76.3–99.5)	75.5 (69.9–81.2)	30.0 (17.9–42.1)	98.4 (97.2–99.7)											0.68
Operator													χ^2^(4) = 6.65	0.156	0.69
Lab tech					85.4 (69.0–100)	74.4 (66.8–82.1)	31.1 (17.5–44.7)	97.8 (94.8–100)							
Nurse					88.0 (74.8–100)	79.1 (73.1–85.1)	35.1 (20.9–49.3)	98.4 (96.6–100)							
Counselor					90.4 (75.4–100)	70.3 (62.3–78.3)	27.5 (15.1–39.7)	98.9 (96.7–100)							
															
Sample type													χ^2^(2) = 12.31	0.002	0.70
Venous									91.1 (80.4–100)	73.1 (66.9–79.3)	28.9 (16.9–40.8)	98.9 (97.3–100)			
Finger prick									80.2 (62.0–98.3)	83.4 (76.8–90.1)	34.2 (19.1–49.3)	97.1 (93.9–100)			

a GLMM generalized through use of a logit link function and binomial distribution with a random intercept for test participant (*n* = 147). PPV and NPV estimates were determined from ordinary logit GLM with cluster robust standard errors. GLMM analyses would not converge reliably. Sens, test sensitivity; Spec, test specificity; PPV, positive predictive value; NPV, negative predictive value; ICC, intraclass correlation coefficient from random intercept GLMM; model A, unadjusted; model B, operator by test interaction and main effect (not shown); model C = Blood sample type by test interaction and main effect (not shown).

b Likelihood ratio (LR) tests comparing nested less-constrained models (B and C) with model A.

### Visitect CD4 test reproducibility.

Importantly, there was no difference detected in the diagnostic performance of the Visitect CD4 between the laboratory technologist and counselor in the clinical lab and the nurse testing at the point of care; LR χ^2^(4) = 7.48, *P* = 0.113; Table 1, model B). For the nurse, who was the only health worker performing the test at the point of care and also on finger-prick blood, the sensitivity was 70.5% (95% CI = 58.3 to 82.7), and the specificity was 86.7% (95% CI = 81.2 to 92.2), whereas the performance by the lab tech and counselor showed a sensitivity of 80.6% (95% CI = 67.9 to 93.3)/specificity of 84.8% (95% CI = 77.8 to 91.8) and a sensitivity of 82.4% (95% CI = 70.1 to 94.8)/specificity of 79.7% (95% CI = 72.0 to 87.4), respectively. A *post hoc* comparison of the effect difference in diagnostic accuracy between testing in the clinic laboratory or at the point-of-care showed no statistically significant effect: Wald χ^2^(1) = 3.70 and *P* = 0.108 (that is, Bonferroni adjusted *–p*^b^*_i_* = *kp_i_*, where *k* is the number of comparisons undertaken; in this instance *k* = 2).

Interobserver reliability between the first readout and second readout of the same test strip by two health workers showed excellent agreement (κ statistic = 100%).

## DISCUSSION

In the era of test-and-treat for all HIV-infected individuals, but with more than a third of people starting ART with advanced HIV disease (22), the specifications and requirements of a true rapid point-of-care CD4 test may need to be further developed and defined (1, 2). An ideal point-of-care CD4 test should be semiquantitative, easily performed by nontechnical and/or nonspecialized health care staff at the bedside, and able to accurately guide decisions related to clinical management and treatment initiation (1, 2).

Our study is the first study to describe the field performance and diagnostic accuracy of the new single-use instrument-free low-cost point-of-care Visitect CD4 test when performed by different health worker cadres. The device showed acceptable pooled sensitivity (81.7%) and specificity (82.6%) at diagnosing CD4 ≤ 350 cells/mm^3^ on venous samples of HIV-infected pregnant women in a public hospital in South Africa, and performance did not differ whether performed by nurses, counselors, or laboratory technicians.

If used for the initiation of co-trimoxazole prophylaxis and preemptive treatment or prioritization of ART for those with a CD4 count of ≤350 cells/mm^3^, the observed sensitivity of Visitect CD4 on venous samples would mean that a fifth of participants with CD4 ≤ 350 cells/mm^3^ were not identified. Performance, however, improved significantly for those with the highest need of ART and co-trimoxazole initiation (23, 24), where the Visitect CD4 correctly identified more than 90% of those with CD4 ≤ 200 cells/mm^3^, although the number of participants was small. These results are also encouraging in that most individuals falsely categorized as having a CD4 count of <350 cells/mm^3^ had a CD4 count close to 350 cells/mm^3^, which reduces the potentially harmful impact of misclassification, particularly in the era of “treat all.”

The Visitect CD4 performance using finger-prick blood samples proved suboptimal (61% sensitivity and 90% specificity) and differed significantly from performance on venous blood. Some (9, 15, 25), though not all (26, 27), studies have alluded to suboptimal performance of CD4 testing using capillary blood. Some staff reported difficulties with obtaining adequate samples from finger pricks and differences in sample collection devices (capillary tube for finger-prick versus micropipette for venous blood) may explain these differences. Given that the nurse performed both a finger prick-based test and a venous blood-based test at the point of care, it is unlikely that the lower performance of the test with finger-prick blood is due to operator error or to it being relatively more difficult to follow test procedures at the bedside than in the laboratory.

Importantly, test performance on venous blood did not alter when conducted by different cadres of health care workers. In our study, conduct of the test by an experienced laboratory technologist was no different from the diagnostic performance of a counselor and a nurse with the same device-specific training. Similar findings were observed in other point-of-care CD4 tests, such as the Pima (28). The somewhat lower sensitivity obtained by the nurse can likely be explained by the fact that the nurse was the only operator doing the tests on the finger-prick samples which we show in model C to have inferior performance.

Before study commencement, operators received an estimated 15 h of training and a 2.5-month practice period, during which operators conducted multiple tests. This proved sufficient, but such training might not be reflective of real-life training opportunities, and minimum training requirements need to be established.

In general, operators liked the simplicity and immediate availability of test results (40 min). A more detailed qualitative exploration of health worker perspectives and the feasibility and acceptability is presented separately (unpublished data). Immediate availability of CD4 test results has important advantages for results communication and enabling urgent linkage to care and ART initiation for those most in need. Point-of-care CD4 testing has been found to be effective in improving patient linkage to care, patient retention in care and timeliness of ART initiation (14, 29). These positive impacts would lead to improvements in treatment effects, the quality of care, and the ultimately quality of life of HIV-infected individuals along the HIV treatment cascade.

All current commercially available point-of-care CD4 testing technologies have one common pitfall: they do require power supply and some level of equipment to perform the test. Such equipment requires initial investments and regular maintenance by a qualified technician. Recent research has shown that inadequate maintenance of test machines, equipment breakdown, commodity stock-out, and lack of appropriate training for health care staff may contribute to unacceptable failure rates, increased machine downtime, and delays in turnaround time of CD4 test results (15, 30). Studies with Pima have reported a wide range of failure rates: with capillary blood this ranged from 2 to 23.3%, and with venous blood in various clinical settings it ranged from 4.8 to 15.2% (15). As a result, there remain serious limitations accessing CD4 testing facilities, particularly in the more remote and rural areas, or in places with lower burden of HIV-infection where investments in a CD4 machine might not be warranted.

A single-use, instrument-free, true point-of-care CD4 test that can perform well when conducted by different health worker cadres and at the point of care, such as the Visitect CD4, could significantly increase patient access to CD4 testing services and respond to the WHO-identified research priority for simplified CD4 cell count testing (31).

### Limitations.

Although use of the mean of the two flow cytometry tests may counter the lack of reliability or repeatability of these tests and better represent the “true” CD4 count of a participant, there are challenges in defining the reference level for a CD4 count. Clearly, inaccuracies introduced by the reference test reduce the sensitivity and specificity of the candidate tests.

In this clinical setting, nearly half the enrolled participants (47%, 72/153) had an average CD4 cell count between 200 and 500 cells/mm^3^, thereby challenging the diagnostic accuracy of any device. Even the gold standard reference flow cytometry had difficulties in six participants, who had discordant results on their duplicate testing of one sample around the cutoff.

The Visitect CD4 test is currently designed to detect patients with a CD4 cell count of ≤350 cells/mm^3^. The development of a Visitect CD4 with lower cutoffs of 100 and/or 200 cells/mm^3^ could be a useful addition to further support evidence-based clinical management.

Although an important target group, the fact that our study only involved pregnant women and a relatively small number of subjects limits the generalizability of our findings. Since pregnancy is associated with a multitude of biological and hormonal changes, it might influence the performance of the test, and this highlights the need for performance data among other patient groups.

Our study was conducted in a referral hospital by well-trained staff with previous experience using lateral flow tests. This might not be a good reflection of performance among health care workers in more remote areas with potentially lower educational background and less training.

### Conclusions.

CD4 measurement has an important role to play in assessing baseline risk of disease progression, particularly for individuals presenting with advanced disease, decisions regarding starting and stopping prophylaxis for opportunistic infections, and prioritization decisions regarding ART initiation in settings where universal treatment is not yet possible. Consistently good performance of the Visitect CD4 on venous blood with different operators, in combination with the fact that no electricity and no instrument is required for conducting the test, shows the potential of this device for decentralization of CD4 testing services in the most resource-constrained settings. Further research is needed to make any conclusions regarding the appropriateness of using finger-prick for CD4 testing. The considerable financial and clinical benefits associated with obtaining a test result at the point of care may outweigh the risks associated with a false-negative result.
